# Occupational exposures and determinants of ultrafine particle concentrations during laser hair removal procedures

**DOI:** 10.1186/s12940-017-0239-z

**Published:** 2017-03-29

**Authors:** Emily J. Eshleman, Mallory LeBlanc, Lisa B. Rokoff, Yinyin Xu, Rui Hu, Kachiu Lee, Gary S. Chuang, Gary Adamkiewicz, Jaime E. Hart

**Affiliations:** 1 Department of Environmental Health, Harvard T.H. Chan School of Public Health, 401 Park Drive, Suite 401 East, Landmark Center, Boston, MA USA; 2 Department of Dermatology, Massachusetts General Hospital, Boston, MA USA; 3 University of California Los Angeles School of Medicine, Los Angeles, CA USA; 4 Channing Division of Network Medicine, Department of Medicine, Brigham and Women’s Hospital and Harvard Medical School, 401 Park Drive, Landmark Center, Boston, MA USA

**Keywords:** Laser, Ultrafine particles, Surgical plume, Laser hair removal, Occupational exposures

## Abstract

**Background:**

Occupational exposures to ultrafine particles in the plume generated during laser hair removal procedures, the most commonly performed light based cosmetic procedure, have not been thoroughly characterized. Acute and chronic exposures to ambient ultrafine particles have been associated with a number of negative respiratory and cardiovascular health effects. Thus, the aim of this study was to measure airborne concentrations of particles in a diameter size range of 10 nm to 1 μm in procedure rooms during laser hair removal procedures.

**Methods:**

TSI Model 3007 Condensation Particle Counters were used to quantify the particle count concentrations in the waiting and procedure rooms of a dermatology office. Particle concentrations were sampled before, during, and after laser hair removal procedures, and characteristics of each procedure were noted by the performing dermatologist.

**Results:**

Twelve procedures were sampled over 4 days. Mean ultrafine particle concentrations in the waiting and procedure rooms were 14,957.4 particles/cm^3^ and 22,916.8 particles/cm^3^ (*p* < 0.0001), respectively. Compared to background ultrafine particle concentrations before the procedure, the mean concentration in the procedure room was 2.89 times greater during the procedure (*p* = 0.009) and 2.09 times greater after the procedure (*p* = 0.007). Duration of procedure (*p* = 0.006), body part (*p* = 0.013), and the use of pre-laser lotion/type of laser (*p* = 0.039), were the most important predictors of ultrafine particle concentrations. Use of a smoke evacuator (a recommended form of local exhaust ventilation) positioned at 30.5 cm from the source, as opposed to the recommended 1–2 in., lowered particle concentrations, but was not a statistically significant predictor (*p* = 0.49).

**Conclusions:**

Laser hair removal procedures can generate high exposures to ultrafine particles for dermatologists and other individuals performing laser hair removal, with exposure varying based on multiple determinants.

## Background

Laser hair removal (LHR) procedures are one of the top five most popular non-invasive procedures performed in the United States, with over 1.1 million treatments conducted in 2014 [1]. Invented in 1998, LHR is based on the theory of extended selective photothermolysis in which the laser device delivers light energy at a specific wavelength and pulse duration targeting melanin in hair follicles [2]. During the procedure, a malodorous and often visible plume is generated.

To date, little is known regarding exposures to LHR plume, although the surgical plume generated from electro-cautery devices is a known occupational hazard for clinicians [3–11]. A recent study by Chuang et al. documented high levels of ultrafine particles (UFP), particles less than 1 μm in aerodynamic diameter, during a LHR procedure [12]. As part of the same study, a total of 63 chemical compounds (including 13 known or suspected carcinogens) were identified in plumes generated during experimental LHR simulations, and 7 chemicals (acetonitrile, acrylonitrile, toluene, ethylbenzene, styrene, propene, and carbon monoxide) were detected in air samples taken during the single LHR procedure [12]. Using smoke evacuators, a form of local exhaust ventilation, at a distance of 1–2 in. from the laser has been recommended to control exposure to LHR plumes. However, even with the use of a smoke evacuator in close proximity (5.0 cm from procedure site), Chuang et al. observed that the concentration of UFP at face level of the laser practitioner during a LHR treatment was 8 times higher than the ambient concentration in the procedure room prior to the treatment, indicating that the smoke evacuator does not fully eliminate the potential for UFP exposures [12].

Chronic exposure to ambient particulate matter (PM) has been linked to detrimental health effects, including lung cancer and other cardiopulmonary diseases [1, 13–16]. The literature on the health effects of UFP is growing rapidly [17]. Acute exposure to ambient UFP has been associated with decreased lung function, decreased heart rate variability, increased inflammation and coagulation in the lungs and peripheral blood, and risk of myocardial infarctions [18–24]. Chronic ambient exposure to UFP has been associated with increased risks of overall and cardiovascular mortality and increased systemic inflammation and oxidative stress [17, 25, 26].

Although there is growing literature on the adverse health effects of ambient exposures to UFP, little is known regarding occupational exposures to UFP during LHR. The goal of this study was to assess the occupational exposure to UFP during LHR in a clinical setting over a series of typical workdays, compare levels of exposure before, during, and after LHR procedures, and identify factors associated with increased UFP exposures.

## Methods

### Study location

The study was performed at the Massachusetts General Hospital Dermatology Laser & Cosmetic Center in Boston, MA. The Center was located in a suite of an office building and the ventilation in the procedure rooms was typical of general office building ventilation systems. Sampling occurred over four typical work days (based on number of and types of procedures). Data collection occurred in the waiting room and two procedure rooms of the clinic for the duration of each work day. Sampling occurred during all scheduled procedures on the selected days. Either an Alexandrite laser (755 nm, pulse duration 3 ms, cryogen-spray cooling, Candela Gentelase. Wayland, MA) or Diode laser (810 nm, pulse duration 30 ms, contact cooling, pre-laser [Lux] lotion application. Lumenis LightSheer. Santa Clara, CA) was used as appropriate for the LHR treatment. The study protocol was reviewed and deemed exempt by the Human Subjects Committee of the Harvard T.H. Chan School of Public Health.

### Data collection

UFP count concentrations (particles/cm^3^) were measured using TSI Model 3007 Condensation Particle Counters (CPC) (Shoreview, MN). To measure ambient UFP concentrations, a CPC was placed at breathing zone height (approximately 1.2 m) in the waiting room prior to the beginning of the first LHR procedure on each day of sampling. The waiting room is connected to the first and second procedure rooms through a corridor, and is 48.8 m from the first procedure room and 76.2 m from the second procedure room. The first and second laser procedure rooms are separated by other non-laser procedure rooms.

Another CPC was moved between two procedure rooms, depending on where treatments were occurring. The CPC was placed on a countertop nearest the procedure table, approximately 1–2 m away from where the procedure was being performed. The locations of the instruments in the procedure rooms were chosen based on the availability of space and the repeatability of the location. The measurements are likely to underestimate actual occupational exposures due to the distance from the plume source.

The CPCs were programmed to record at 1-s intervals. For each procedure, the dermatologist documented the start and stop time of the procedure, the body part undergoing LHR (abdomen, back, bikini, face, legs or underarm), laser type, fluence, pulse duration, number of laser pulses, use of pre-laser (Lux) lotion, and whether or not a smoke evacuator (Buffalo PlumeSafe Turbo) was used during the procedure. If a smoke evacuator was used, it was held approximately 30.5 cm away from the treatment area, as was standard practice at this facility to avoid interfering with the LHR procedure.

### Quality assurance and quality control procedures

All instruments were collocated in the waiting room for at least 5 min. The CPCs were deemed to have an acceptable level of precision if the average percent difference in UFP concentrations between the collocated instruments was less than 25%. Pre- and post- sampling flow rates were measured and recorded for all instruments and a 5% or less deviation from the manufacturer specified flow rate of 0.7 L/min was deemed acceptable. Any procedure that did not meet the above quality control criteria was excluded from the analyses.

### Statistical analysis

All statistical analyses were performed using SAS 9.4, SAS Institute Inc. NC. The 1-s interval data was collapsed into 1-min interval data points for further analysis. Before and after procedure UFP concentrations were defined as the average concentrations during the time periods before and after each procedure, with the time period equal to the corresponding procedure duration. For example, if the procedure length was three minutes, the before and after concentrations would be the average concentrations over the three minutes prior to the procedure and the three minutes following the procedure, respectively. Therefore, three exposure metrics for UFP concentration over time were available for each procedure.

To account for correlation within the UFP data, Wilcoxon signed-rank tests were used to assess differences in the distributions of UFP concentrations between the waiting room and the procedure rooms overall during the 4 days of sampling. Longitudinal mixed models with an unstructured variance-covariance matrix, adjusted for location (procedure room or waiting room), were used to analyze differences in the mean concentrations of UFP over time (before, during, and after the procedures).

To determine predictors of average UFP concentrations during LHR procedures, univariate Generalized Linear Regression models were developed. The potential predictors examined included body part (face, bikini, back, legs, or abdomen), duration of the procedure (in minutes), laser and pre-laser lotion use (Diode laser with Lux lotion vs. Alexandrite laser without Lux lotion), and the use of a smoke evacuator (yes/no). Due to limited sample size, each covariate, or set of covariates was included in separate models. A two-sided *p*-value of 0.05 was used to determine statistical significance and we examined the normality of model residuals to determine if the model using untransformed UFP values were appropriate.

## Results

A total of 17 laser hair removal treatments were sampled over 4 days. The total sample time was 1,087 min in the procedure rooms and 1,105 min in the waiting room. Four procedures were excluded for failing the flow rate quality control checks and an additional procedure was excluded because the instrument battery died before the procedure was completed. After these exclusions, 12 procedures (70.6%) were available for analyses. Procedures ranged from 1 to 34 min, and included abdomen, face, leg, bikini, underarm and back treatments (Table 1).

**Table 1:** Characteristics of all sampled procedures and of sampled procedures included in the final data analysis

	All procedures			Procedures included in analysis			
		Duration (min)			Duration (min)		Average ± Standard Deviation Ultrafine Particle Concentration
Characteristic	*N*	Average	Range	*N*	Average	Range	(Particles/cm^3^)
Body part							
Face	4	6	(2–14)	3	6	(2–14)	20,334.32 ± 2,540.94
Bikini	5	12	(4–34)	3	15	(4–34)	41,563.06 ± 16,179.49
Back	2	8	(1–14)	2	8	(1–14)	44,007.44 ± 28,483.18
Legs	2	20	(19–21)	2	20	(19–21)	105,417.27 ± 17,379.98
Underarms	3	5	(2–9)	1	4	–	50,862.5
Abdomen	1	5	–	1	5	–	5,820.04
Lotion used	11	64.71%		6	50.00%		
Smoke evacuator used	12	70.59%		8	66.67%		

**Fig. 1:**
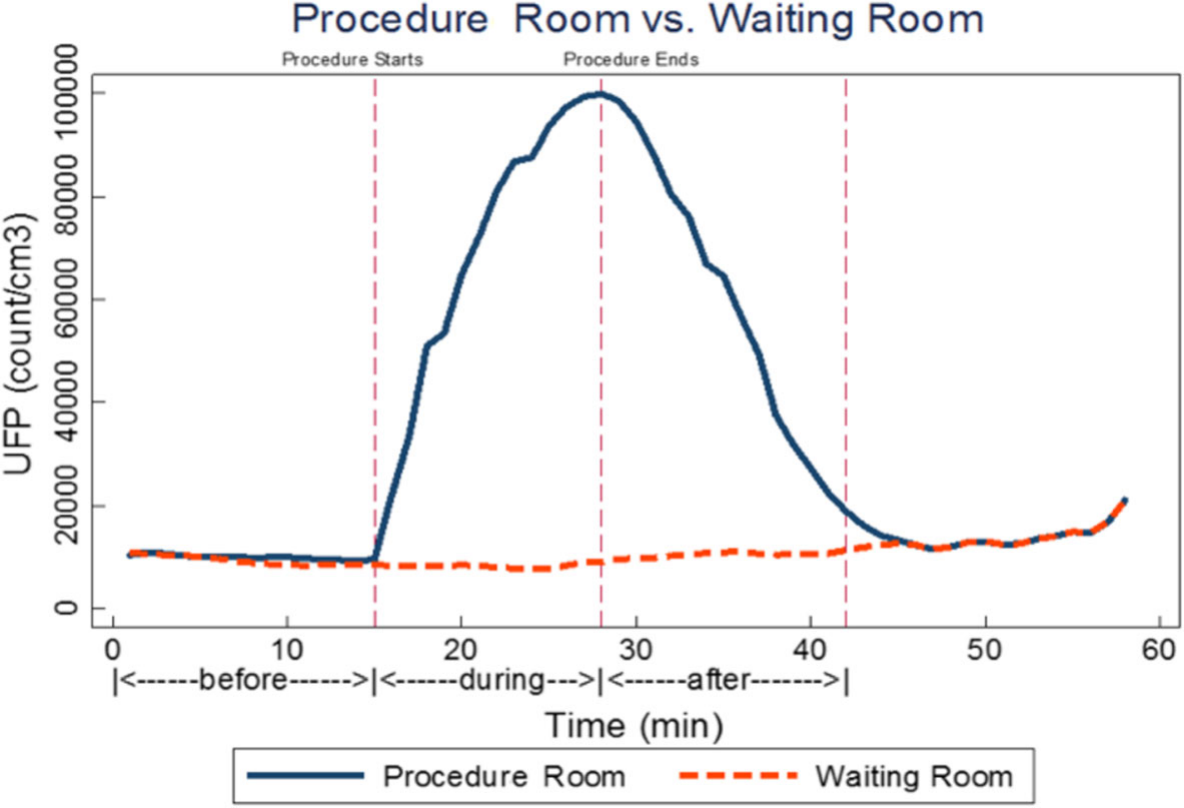
Ultrafine particle concentrations (particles/cm^3^) in a procedure room and waiting room during a typical procedure laser hair removal procedure

The distributions of UFP concentrations in the waiting and procedure rooms overall and by time period are shown in Table 2. The mean UFP concentration in the waiting room overall (14,957.4 particles/cm^3^) was lower than levels in the procedure rooms (22,916.8 particles/cm^3^; *p* < 0.0001). UFP concentration profiles in the waiting and procedure rooms during a typical procedure are shown in Fig. 1.

**Table 2:** UFP concentrations (particles/cm^3^) in the waiting and procedure rooms overall, and before, during, and after procedures

Location	*N*	Mean	SD	Median	IQR	95^th^ percentile	99^th^ percentile
Waiting room							
Overall	1105	14,957.4	6,246.8	14,704	3,974–31,700	26,154	29,711
Before LHR	130	14,997.1	4,911.6	15,747	10,492–17,373	24,576	25,060
During LHR	138	15,597.1	5,144.8	16,344	10,787–19,433	23,713	24,712
After LHR	126	16,350.0	4,957.0	16,856	12,112–19,269	24,847	29,189
Procedure room							
Overall	1087	22,916.8	24,853.5	15,970	4,882–206,674	72,491	146,442
Before LHR	130	14,332.9	4798.1	15,035	10,170–16,458	24,420	26,211
During LHR	138	55,046.1	47,761.2	37,469	17,671–78,690	158,819	187,185
After LHR	126	36,551.4	24,623.4	27,534	18,876–47,504	87,263	116,617

UFP concentrations increased rapidly in the procedure room during a LHR treatment, peaked at the end of the procedure, and decreased steadily during the after-procedure period. However, the UFP concentrations did not return to the pre-treatment concentrations by the end of the “after treatment” period. There was no noticeable increase in UFP concentrations in the waiting room during the treatments.

In mixed models, compared to levels before procedures, the average UFP concentrations in the procedure room were 2.89 times higher during procedures (*p* = 0.009) and 2.09 times higher (*p* = 0.007) after the procedures.

Body part receiving the treatment was a statistically significant predictor of UFP exposure (*p* = 0.013). Body part predicted 86.7% of the variability in UFP levels. Compared to LHR procedures on underarms, UFP concentrations during leg procedures were statistically significantly higher (Table 3), while all other body parts were lower. Duration of the procedure was also a statistically significant predictor of UFP concentration, with each one minute increase in duration associated with an increase of 3,643.4 particles/cm^3^ (*p* = 0.006), after excluding one outlier procedure (34-min bikini treatment), although overall procedure duration only predicted 58.8% of the variability in UFP concentrations. Procedures using the Alexandrite laser had statistically significantly higher UFP concentrations (38,614.2 particles/cm^3^, *p* = 0.039) compared to procedures using the Diode laser and the Lux lotion. Use of the smoke evacuator at 30.5 cm away from the skin decreased concentrations by 14,781.6 particles/cm^3^ on average, but this decrease was not statistically significant (*p* = 0.498) and described little of the variability in UFP levels (4.6%).

**Table 3:** Results of Univariate Models Examining Predictors of UFP Concentrations During LHR Procedures

Predictor	Effect estimate (Particles/cm^3^)	Standard error	*p*-value	*R* ^2^
Body part				0.867
Abdomen	−45,042.5	23,450.8	0.103	
Back	−6,855.1	20,309.0	0.747	
Bikini	−9,299.4	19,147.5	0.644	
Face	−30,528.2	19,147.5	0.162	
Legs	54,554.8	20,309.0	0.036	
Underarm	0.0 Ref.			
Laser and lotion use				0.361
Alexandrite laser without lotion	38,614.2	16,236.9	0.039	
Diode laser with lotion	0.0 Ref.			
Smoke evacuator use				0.046
No evacuator use	14,781.6	21,035.3	0.498	
Evacuator Use	0.0 Ref.			
Procedure duration^a^				0.588
1 min increase	3,643.4	1,016.6	0.006	

Each predictor, or set or predictors was assessed in separate generalized linear regression models

^a^Excluding a 34-min bikini procedure

## Discussion

In the largest study of occupational exposure to UFP from LHR to date, we observed statistically significant elevations in UFP concentrations in procedure rooms during and after LHR, compared to concentrations before treatments and concurrent measurements in the waiting room. A number of procedure characteristics were associated with higher UFP levels during LHR. These included the body part undergoing treatment, laser type and use of Lux lotion, and procedure duration. Use of a smoke evacuator at 30.5 cm away during the procedure was not a statistically significant predictor of UFP concentrations, although there was evidence of decreased particle counts when in use.

Duration of procedure was the most statistically significant predictor of average UFP concentrations and is a desirable characteristic to consider, as it can be easily measured in both future studies and in practice. Body part of the procedure was another statistically significant predictor of the average concentration of UFP, but procedures by body part are highly variable by patient, due to varying density of hair, and are therefore harder to generalize. The combined use of pre-laser Lux lotion and the diode laser reduced emissions by up to 60.0% compared to procedures using the Alexandrite laser. The pre-laser lotion was used with the contact cooling diode laser to facilitate cooling and prevent charring. In contrast, no lotion was used with the Alexandrite laser, which is equipped with a cryogen Dynamic Cooling Device (DCD). Two main factors may contribute to higher UFP count associated with Alexandrite laser hair removal. First, the shorter pulse duration of the Alexandrite laser (3 ms) results in a higher energy delivered than the diode laser (30 ms), potentially causing increased plume. The Alexandrite was used at a frequency of 1.5 Hz and the diode was used at a frequency of 1 Hz. In addition, the cryogen spray incorporated into the Alexandrite laser produces a sudden airflow which likely disperses the UFP, whereas contact cooling in diode laser produces minimal airflow. Moreover, the use of pre-laser lotion may prevent thermal charring of the surface hair shaft and trap the UFP produced during LHR.

Surgical smoke is a known occupational hazard containing UFP [3–11, 27]. An estimated 500,000 healthcare workers are exposed to laser generated surgical smoke each year [27]. However, there are no regulatory occupational standards under the Occupational Safety and Health Administration (OSHA) specifically for surgical smoke, only guidelines issued by the National Institute for Occupational Safety and Health (NIOSH) and the American National Standards Institute (ANSI) (standards Z136.3-2014 and Z136.1-2007) [3, 28, 29]. NIOSH recommends the use of local exhaust ventilation in the form of smoke evacuators with a suction vacuum pump, hose, inlet nozzle, and a high efficiency particulate air (HEPA) filter to reduce the UFP concentration. The recommendations state that the evacuator nozzle should be placed within 2 in. of the plume in order to effectively capture the particles [29]. During the sampled procedures, the use of the smoke evacuator did lead to decreased average UFP concentrations, although not a statistically significant reduction in particles. However, to reduce impact to the LHR workflow, the laser practitioners in this clinic commonly kept the smoke evacuator further than the NIOSH recommended 2 in. from the operating field (on average about 30.5 cm). Thus, the data collected in this study did not fully evaluate the effectiveness of a smoke evacuator to reduce LHR particulates when used as recommended by NIOSH.

We also observed that particle clearance following the procedure is steady but not immediate. As in the example in Fig. 1, it took approximately the same amount of time after a procedure for the UFP concentrations to approach the pre-procedure levels as it did to generate them (i.e. the procedure duration). This finding suggests that there is potential for UFP exposures to occur after the LHR procedure is concluded. Laser practitioners or medical assistants may be present in the room during this post-procedure period. It is also possible (although this did not occur during our sampling) that a subsequent procedure could begin in the same room before there was complete clearance, which could lead to higher peak UFP concentrations. Although we saw a steady decrease in UFP concentrations post-procedure, we did not record a complete return to background concentrations during any of our sampling periods.

In the only other study of UFP exposures during LHR, Chuang et al. reported a 8-fold increase in average UFP concentrations in the procedure room during a treatment with the use of a smoke evacuator compared baseline level in the room prior to any procedure [12], which is consistent with our findings that peak UFP concentrations in the procedure room were 6.7 times greater than our background, waiting room concentrations. The median levels in the procedure room during treatments were comparable to median levels observed in urban backgrounds in the Boston metropolitan area, but were approximately half the median levels observed within 400 m of major roadways [30]. To date, no threshold has been observed in studies of the adverse health effects of UFP exposures. The UFP levels observed in this study were more than two times higher than concentrations at which area-level UFP exposure has been associated with oxidative stress, potentially producing long-term health hazards [31]. Therefore, although not a focus of the current study, it is concerning that the current levels of UFP exposure experienced during LHR procedures may have adverse health effects.

Our study has several limitations. First, although we did sample a number of days, we only had a small number of procedures available to assess. Small sample size prevented powering multivariable regression that included all potential predictors of UFP, or the ability to assess interactions between predictors. Therefore, we are unable to evaluate sets of factors (other than laser type and lotion use) that may co-occur in practice. Further investigations are warranted because of the potential for confounding between covariates (i.e. duration of procedure and body part) that we were unable to assess. It is likely that other factors, such as specific clinician performing a procedure, or the coarseness of hair can play a role in the amount of laser plume generated in each procedure, but this was not accounted for in our study. We were also limited in our ability to directly assess occupational exposures, because the CPCs were located outside of the dermatologists’ breathing zones to prevent impacting clinical workflow, which differs from Chuang et al., where the CPCs were placed at the level of the practitioner and the patient. Additionally, we were not able to fully evaluate the effectiveness of the recommended use of a smoke evacuator, since it was standard practice for the laser practitioners that participated in this study to use the smoke evacuator at approximately 30.5 cm from the source, which proved to not provide a significant reduction in UFP concentrations. Lastly, we are unable to generalize our results to the full range of locations where LHR is performed, given different clinical practices across clinics and beauty salons/spas and potential differences in the distribution of exposure predictors. While our study was aimed at understanding potential exposures in a typical clinical setting, a randomized study design would be best suited to isolating the effects of specific procedural factors on exposures. Finally, our study cannot inform the relative benefit of various approaches to reduce exposures, such as proper evacuator use or increases in spot or general ventilation. However, based on first principles, we believe that these approaches would achieve some degree of exposure reduction.

This is the first study that we know of to look at UFP concentrations across multiple LHR procedures. Since the number of LHR appointments can differ greatly from day to day, a 1-day sample could potentially limit a study’s ability to capture a dermatologist’s average workday exposure. Since our sample was a relatively larger (N = 12) procedure sample size compared to the only existing study (N = 1), we were able to assess changes in particle concentrations before, during, and after each procedure and assess the impact of procedure characteristics on average UFP concentrations.

## Conclusions

The results of this study demonstrate that LHR procedures have the potential to introduce UFP exposures that are well above ambient concentrations, and that there are easily measured key drivers of exposure. Results of our exploratory analysis indicate that duration of the procedure is a strong indicator of UFP concentrations, but other covariates and potential confounders should be considered further in future studies. Our results suggest that utilizing a smoke evacuator outside of the recommended range of 2 in. from the source provides minimal protection for laser practitioners. Laser practitioners should utilize a smoke evacuator during every procedure and follow the NIOSH recommended work practices. The significant decrease in emissions seen with the use of pre-laser lotion warrants further research to determine if pre-laser lotion can be used to mitigate UFP exposures or if these findings were a result of laser type. Potential mitigation strategies would need to be tested for compliance and efficacy in a variety of procedure room configurations and for a variety of procedures to ensure wide applicability and effectiveness.

## Abbreviations

CPC: Condensation Particle Counter
LHR: Laser hair removal
PM: Particulate matter
UFP: Ultrafine particles
